# Amiloride but Not Memantine Reduces Neurodegeneration, Seizures and Myoclonic Jerks in Rats with Cardiac Arrest-Induced Global Cerebral Hypoxia and Reperfusion

**DOI:** 10.1371/journal.pone.0060309

**Published:** 2013-04-08

**Authors:** Kwok Keung Tai, Daniel D. Truong

**Affiliations:** 1 The Parkinson’s and Movement Disorder Research Laboratory, Long Beach Memorial Medical Center, Long Beach, California, United States of America; 2 The Parkinson’s and Movement Disorder Institute, Fountain Valley, California, United States of America; The Ohio State University, United States of America

## Abstract

It has been reported that both activation of N-methyl-D-aspartate receptors and acid-sensing ion channels during cerebral ischemic insult contributed to brain injury. But which of these two molecular targets plays a more pivotal role in hypoxia-induced brain injury during ischemia is not known. In this study, the neuroprotective effects of an acid-sensing cation channel blocker and an N-methyl-D-aspartate receptor blocker were evaluated in a rat model of cardiac arrest-induced cerebral hypoxia. We found that intracisternal injection of amiloride, an acid-sensing ion channel blocker, dose-dependently reduced cerebral hypoxia-induced neurodegeneration, seizures, and audiogenic myoclonic jerks. In contrast, intracisternal injection of memantine, a selective uncompetitive N-methyl-D-aspartate receptor blocker, had no significant effect on cerebral hypoxia-induced neurodegeneration, seizure and audiogenic myoclonic jerks. Intracisternal injection of zoniporide, a specific sodium-hydrogen exchanger inhibitor, before cardiac arrest-induced cerebral hypoxia, also did not reduce cerebral hypoxia-induced neurodegeneration, seizures and myoclonic jerks. These results suggest that acid-sensing ion channels play a more pivotal role than N-methyl-D-aspartate receptors in mediating cerebral hypoxia-induced brain injury during ischemic insult.

## Introduction

A continuous supply of oxygen and nutrients to the brain is vital for maintaining its normal functioning. Hypoxia-induced permanent neuronal damage in the brain can occur in either focal ischemia during ischemic stroke, traumatic insults, cerebral hemorrhages or global ischemia during cardiac arrest as a result of heart diseases, heart attack as well as other traumatic conditions such as respiratory arrest, electrocution, drowning, choking and traumas. To understand the mechanism underlying hypoxia-induced brain injury is crucial to improve neurological morbidity and lessen mortality in ischemic stroke patients and in cardiac arrest survivors. Excessive stimulation of N-methyl-D-aspartate (NMDA) receptors by excitatory neurotransmitter, glutamate, during ischemic insult is believed to play a contributing role in ischemic and traumatic brain injury. This theory was first established by the observations that overdose of systemic glutamate destroyed hypothalamic nuclei in mice and infant primates [1], [2] and high concentrations of glutamate that induced neuronal death *in vitro* [3] was also detected in rodent brain during ischemia [4]. It was subsequently demonstrated in *in vivo* models that glutamate induced neuronal death via activation of NMDA receptors [5], [6]. However, results from clinical trials of the NMDA receptor antagonists in stroke and severe head injury patients are disappointing [7], [8], [9]. Failure to translate the results from *in vitro* and *in vivo* studies to clinical studies in humans casts doubt on the role of NMDA receptor-mediated excitotoxicity in cerebral ischemic injury [10].

Acidosis is one of the factors aggravates ischemic brain injury during cerebral ischemia [11]. Results from studies by others in the rat and mice stroke models induced by transient middle cerebral artery occlusion [12] suggested that activation of acid-sensing channels (ASICs) during cerebral ischemia contributed to ischemic brain injury. Activation of the ASICs by the lower extracellular pH as a result of acidosis allows influx of calcium ions through the channels into the neuronal cells, the intracellular calcium-overload induces cell injury and cell death. But which of these two molecular targets play a more important role in hypoxia-induced brain injury during ischemia have not been directly compared and evaluated in animal model of global cerebral hypoxia. In the present study, we compared the neuroprotective, anti-seizure and anti-myoclonus activities of amiloride, an ASICs blocker; memantine, an uncompetitive NMDA receptor blocker and zoniporide, a specific sodium-hydrogen exchanger inhibitor [13], in a rat model of cardiac arrest-induced global cerebral hypoxia and reperfusion which is considered to be the most clinically relevant model for the most dangerous form of global cerebral ischemia encountered in clinical situations [14]. We found that amiloride but not memantine or zoniporide protected against cardiac arrest-induced cerebral hypoxic neurodegeneration, seizures and myoclonic jerks. The results demonstrated that ASICs play a more important role than NMDA receptors in cerebral hypoxia-induced brain injury.

## Materials and Methods

### Intracisternal Injection

Male Sprague-Dawley rats of 220–240 grams were purchased from Charles River Laboratories. Intracisternal injection of 5 µl saline or 0.5, 1.5 and 5 nmole of amiloride, memantine, zoniporide to the animals before cardiac arrest were performed as previously described [15], [16]. Because amiloride is also known to inhibit sodium-hydrogen exchanger (NHE-1), the neuroprotective effect of NHE-1 inhibition by intracisternal injection of zoniporide (0.5, 1.5 and 5 nmole), a specific NHE-1 inhibitor, was also evaluated. Rats were anesthetized with intraperitoneal injection of ketamine (50 mg/kg) and xylazine (5 mg/kg). Sodium pentobarbital (Nembutal) (40 mg/kg) supplemented with a single subcutaneous injection of buprenorphine (0.05 mg/kg) were used to anesthetize the rats when investigated whether the neuroprotective effects of the drugs can be reproduced with anesthetic agent without NMDA receptor blocking activity. Toe pinch stimuli were applied to the animals to ensure adequate level of anesthesia was attained before proceeding to intracisternal injection. Drug was administered intracisternally 4 hours before the experimental cardiac arrest. Except otherwise stated, all chemicals were purchased from Sigma-Aldrich Chemicals (St. Louis, Missouri).

### Cardiac Arrest-Induced Global Cerebral Hypoxia and Reperfusion

A rat model of cardiac arrest-induced cerebral hypoxia previously described was used in the present study [17], [18], [19]. The procedures for generation of this animal model are as follows. The left femoral artery and vein of the anesthetized rat were catheterized to monitor blood pressure and administration of epinephrine to initiate resuscitation. Cerebral hypoxia was induced by mechanical compression of the aorta between the body cavity and an L-shaped loop inserted underneath the major cardiac vessels through the rat thoracic cavity. The arterial blood pressure was reduced to 0–5 mmHg during cardiac arrest. After 8 minutes and 30 seconds, resuscitation began by intravenous injection of 10 µg/kg epinephrine and 4 mEq/kg sodium bicarbonate, followed by manual thoracic compressions for 1–2 minutes until the systemic blood pressure of the animal was returned to normal preoperative level. The animals were monitored for spontaneous seizures by video for three days. They were also subjected to quantitative evaluation of audiogenic myoclonic jerks as previously described [17], [20], [21]. Frozen rat brain sections were collected for evaluation the degree of cerebral hypoxia-induced neurodegeneration using Fluoro-Jade B (FJ) staining.

### Fluoro-Jade B Staining

The evaluation of cerebral hypoxia-induced neurodegeneration using FJ staining was performed as previously described [16], [22], [23]. FJ was purchased from Millipore (Temecula, California). Degenerating neurons were stained with FJ and were visualized using a fluorescence microscope as glowing green cells. The number of FJ-positive degenerating neurons was manually counted under a fluorescence microscope. Brain sections as close as possible to the landmarks of Bregma −3.3 mm and interaural 5.7 mm were identified and selected for counting the number of FJ-positive degenerating neurons in the hippocampus and the thalamic reticular nuclei (TRN). Brain sections at Bregma −12.8 mm and interaural −3.8 mm were chosen for comparison of the number of FJ-positive degenerating neurons in the cerebellum between groups.

The animals use in this study was approved by the IACUC of the Long Beach Memorial Medical Center.

### Statistical Analysis

The number of FJ-positive degenerating neurons between the groups and the myoclonus score at the corresponding time point between the groups were compared using unpaired Student’s *t*-test. The number of animals that developed seizures between the groups were compared using Fisher’s exact test. A *p*<0.05 was considered as statistically significant.

## Results

**Figure 1:**
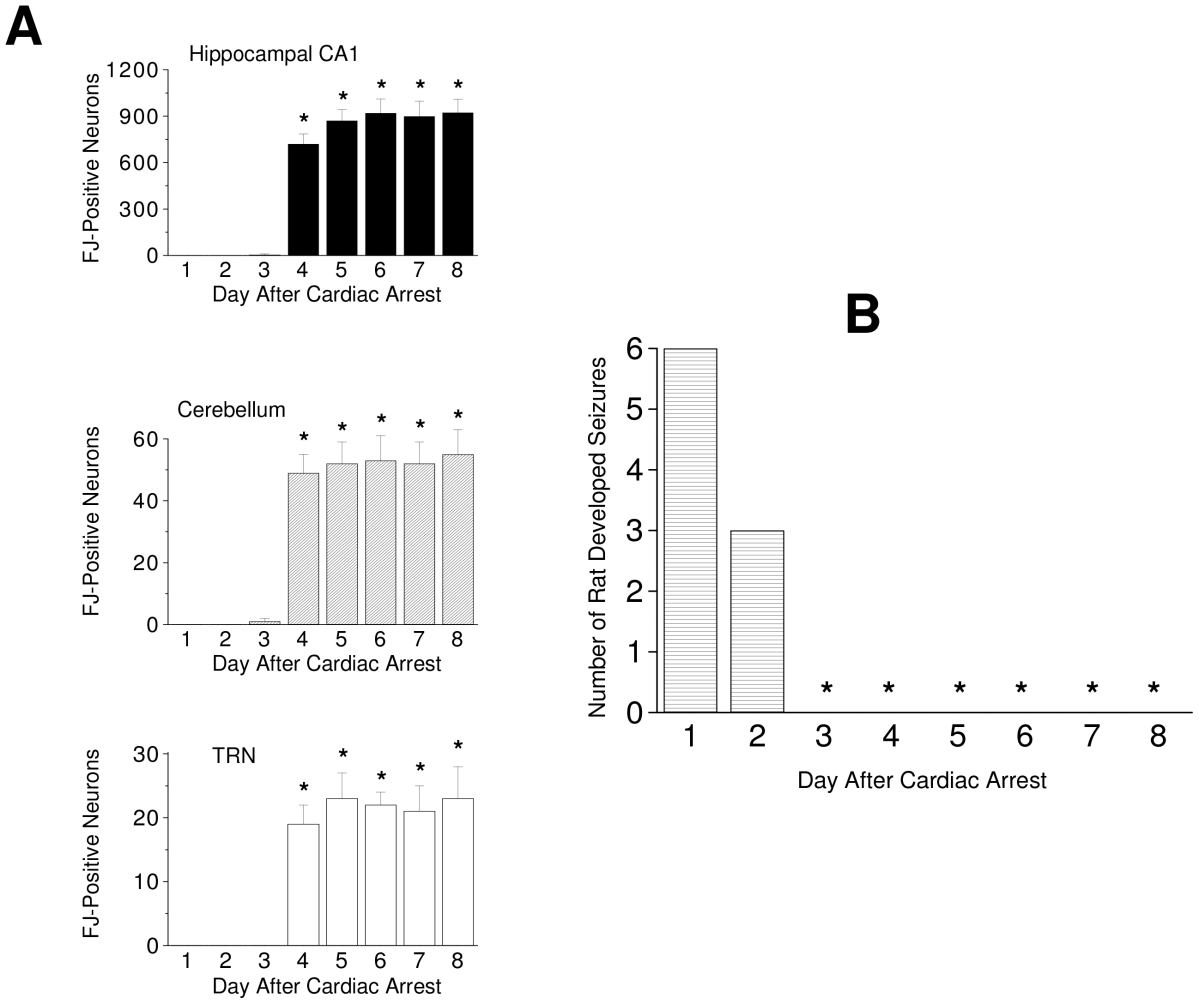
The time course of cardiac arrest-induced cerebral hypoxic neurodegeneration and seizures. (A) The number of FJ-positive degenerating neurons in the hippocampal CA1, the cerebellum, and the TRN of the saline-injected rats subjected to cardiac arrest-induced cerebral hypoxia. (B) The number of animals that developed seizures after cardiac arrest-induced cerebral hypoxia. * (*p*<0.05) indicates significantly different from that of day 1 after cardiac arrest-induced cerebral hypoxia. Values are mean ± S.D., n = 6.

Cardiac arrest-induced global cerebral hypoxia caused neurodegeneration in the CA1 region of the hippocampus, the Purkinje cell layer of the cerebellum and the TRN. The FJ-positive degenerating neurons did not appear until the fourth day after the cardiac arrest in saline-injected rats (Fig. 1A) indicating the global cerebral hypoxia-induced neurodegeneration occurred in a delayed manner. Six out of the six rats that subjected to cardiac arrest-induced cerebral hypoxia developed seizures on the first day after the cardiac arrest. Seizures subsided on day two after cardiac arrest-induced cerebral hypoxia, only three out of the six rats developed seizures (Fig. 1B). No seizure was observed in the posthypoxic rats from day three after cardiac arrest-induced cerebral hypoxia (*p*<0.05) (Fig. 1B).

**Figure 2:**
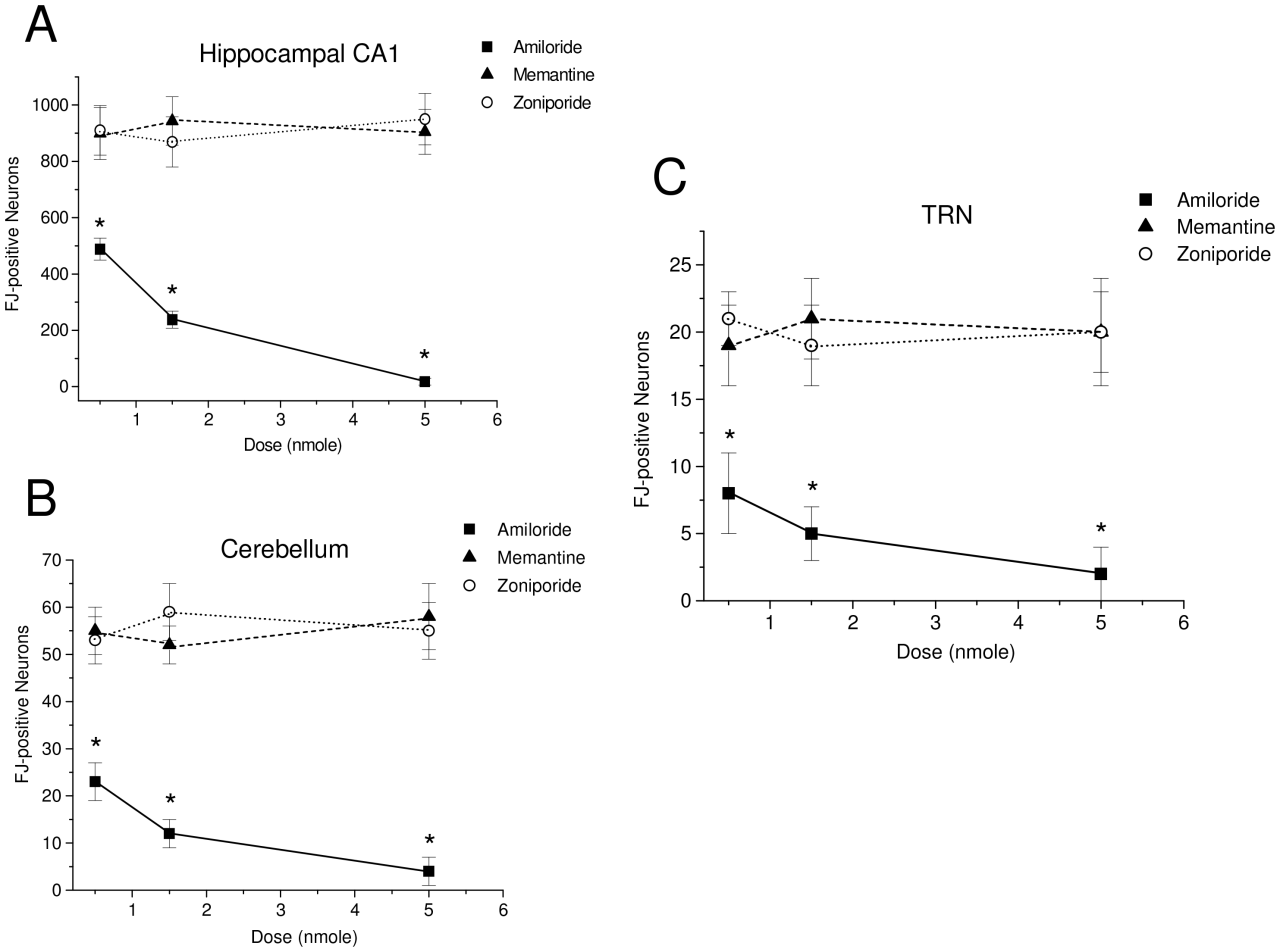
Amiloride but not memantine or zoniporide dose-dependently reduced cardiac arrest-induced cerebral hypoxic neurodegeneration. (A) The number of FJ-positive degenerating neurons in the hippocampal CA1, the cerebellum and the TRN of the rats that received 0.5, 1.5 and 5 nmole of amiloride, memantine or zoniporide before cardiac arrest-induced cerebral hypoxia. * (*p*<0.05) indicates significantly different from that of the saline-, memantine- or zoniporide-injected groups. Values are mean ± S.D., n = 6.

**Figure 3:**
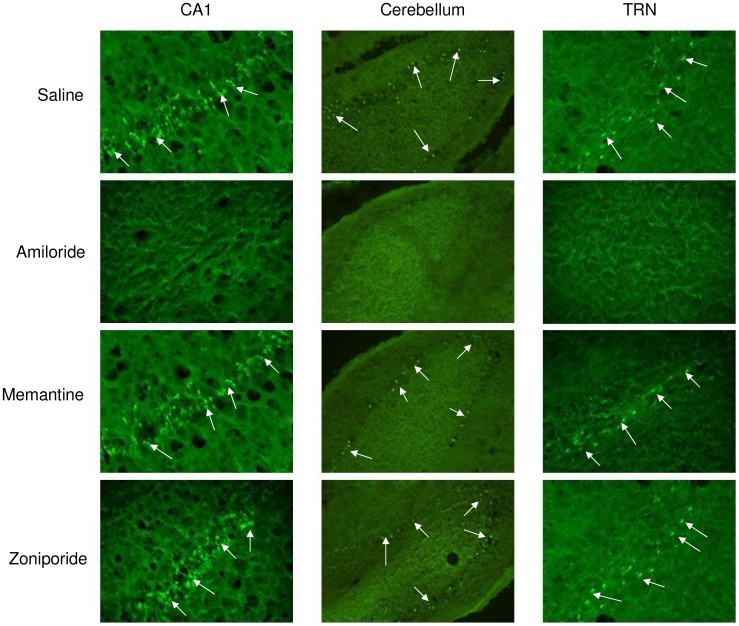
Intracisternal injection of amiloride but not memantine or zoniporide reduces cardiac arrest-induced cerebral hypoxic neurodegeneration. FJ staining of representative coronal brain sections showing the hippocampal CA1, the cerebellum, and the TRN of the rats that received intracisternal administration of saline or 5 nmole of amiloride, memantine or zoniporide before cardiac arrest-induced cerebral hypoxia. The arrows indicate some of the FJ-positive degenerating neurons in the rat brain sections. Pictures were taken at 20X for the hippocampal CA1, the TRN and at 10X for the cerebellum.

The number of FJ-positive degenerating neurons in the hippocampal CA1 (Fig. 2A; Fig. 3), the cerebellum (Fig. 2B; Fig. 3) and the TRN (Fig. 2C; Fig. 3), of the amiloride-treated rats are significantly fewer than that of the saline-injected control group (Fig. 1A), memantine- or zoniporide-treated group. Amiloride-induced neuroprotection occurred in a dose-dependent manner with a progressive reduction in neurodegeneration in these areas of the brain in relation to an increase in its dosage.

In contrast to amiloride, the number of FJ-positive degenerating neurons in the hippocampal CA1 (Fig. 2A), the cerebellum (Fig. 2B), the TRN (Fig. 2C) of the memantine- or zoniporide-treated rats are not significantly different from that of the saline-injected control group (Fig. 1A) indicating that either memantine or zoniporide has no effect on cerebral hypoxia-induced neurodegeneration. In addition, the number of FJ-positive degenerating neurons in the rats that received between 0.5, 1.5 and 5 nmole of memantine or zoniporide in these three areas of the brain are not significantly different from each other. These results suggest that amiloride but not memantine or zoniporide is neuroprotective against cerebral hypoxia-induced neurodegeneration and that the neuroprotective effect of amiloride may result from blockade of ASICs not by inhibition of NHE-1.

Six out of the six animals subjected to cardiac arrest-induced cerebral hypoxia developed seizures day one after the operation. Amiloride reduced the number of posthypoxic rats that developed seizures, only two out of the six posthypoxic rats developed seizures in the amiloride-treated group (Fig. 3A). As for the memantine- or zoniporide-treated group, six out of the six posthypoxic rats developed seizures indicating both memantine and zoniporide do not have anticonvulsant activity against cerebral hypoxia-induced seizures.

**Figure 4:**
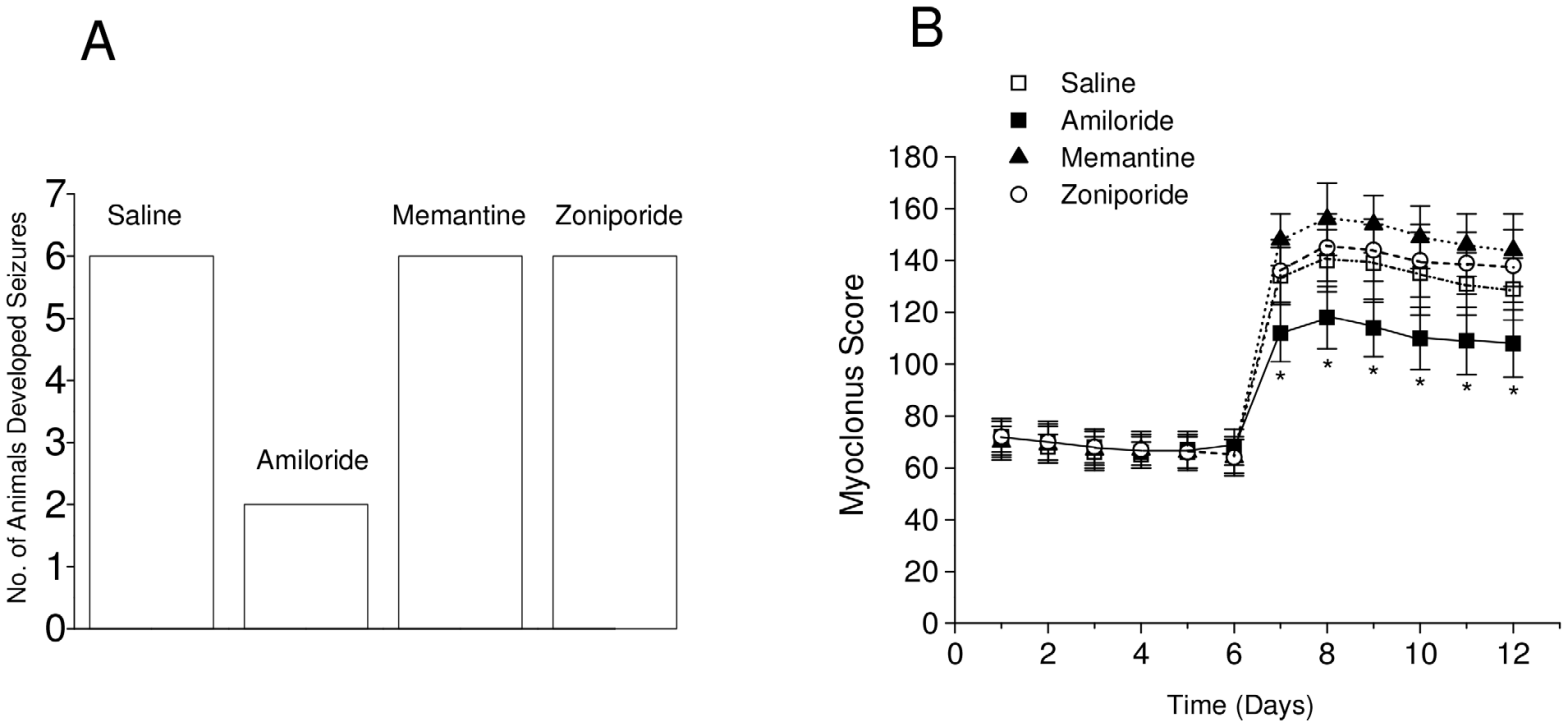
Amiloride but not memantine or zoniporide reduces the number of posthypoxic rats developed seizures and the severity of audiogenic-myoclonic jerks. (A) The number of the rats in the saline-injected, amiloride-, memantine- or zoniporide-treated group that developed seizures after cardiac arrest-induced cerebral hypoxia. (B) The myoclonus scores of the rats before and after cardiac arrest-induced cerebral hypoxia. Cardiac arrest-induced cerebral hypoxia was introduced on day 6. Rats were received intracisternal administration of 5 nmole of amiloride, memantine, zoniporide or 5 µl saline before cardiac arrest. * (*p*<0.05) indicate significantly different from that of the memantine-, zoniporide- or saline-injected control group at the corresponding time point. Values are mean ± S.D., n = 6.

The effects of amiloride, memantine or zoniporide on audiogenic myoclonic jerks of the posthypoxic rats were also evaluated. Cardiac arrest-induced cerebral hypoxia surgical operation was performed on day 6 (Fig. 4B). Rats of the saline-injected control group have a mean myoclonus score of 67±6 (n = 6) before cardiac arrest. Saline-injected control rats exhibited myoclonic jerks in response to auditory stimuli, they have mean myoclonus scores of 134±11, 140±12, 139±15, 135±16, 131±12, 129±12 (n = 6) over the six consecutive days following cardiac arrest. Rats treated with amiloride before cardiac arrest have respective mean myoclonus scores of 112±11, 118±12, 114±11, 110±12, 109±13, 108±13 (n = 6) over the six days after cardiac arrest, each of these scores is significantly lower than (*p*<0.05) that of the saline-injected control group at the corresponding time point (Fig. 4B). This result suggests that amiloride reduced audiogenic myoclonic jerks in the posthypoxic rats. In contrast, rats treated with memantine before cardiac arrest have respective mean myoclonus scores of 148±10, 156±14, 154±11, 149±12, 146±12, 144±14 (n = 6) over the six consecutive days after the cardiac arrest (Fig. 4B). The memantine-treated group seems to have a slightly higher myoclonus scores in the corresponding time points than that of the saline-injected hypoxic animals. For the zoniporide-treated group, the posthypoxic rats have respective myoclonus scores of 136±12, 145±13, 144±12, 140±14, 139±12, 138±14 (n = 6) recorded over the six days after cardiac arrest. The myoclonus scores of the memantine- or zoniporide-treated group are not significantly different that of the saline-injected hypoxic animals. These results suggest that either memantine- or zoniporide-treatment has no effect on the audiogenic myoclonic jerks in the posthypoxic rats.

**Figure 5:**
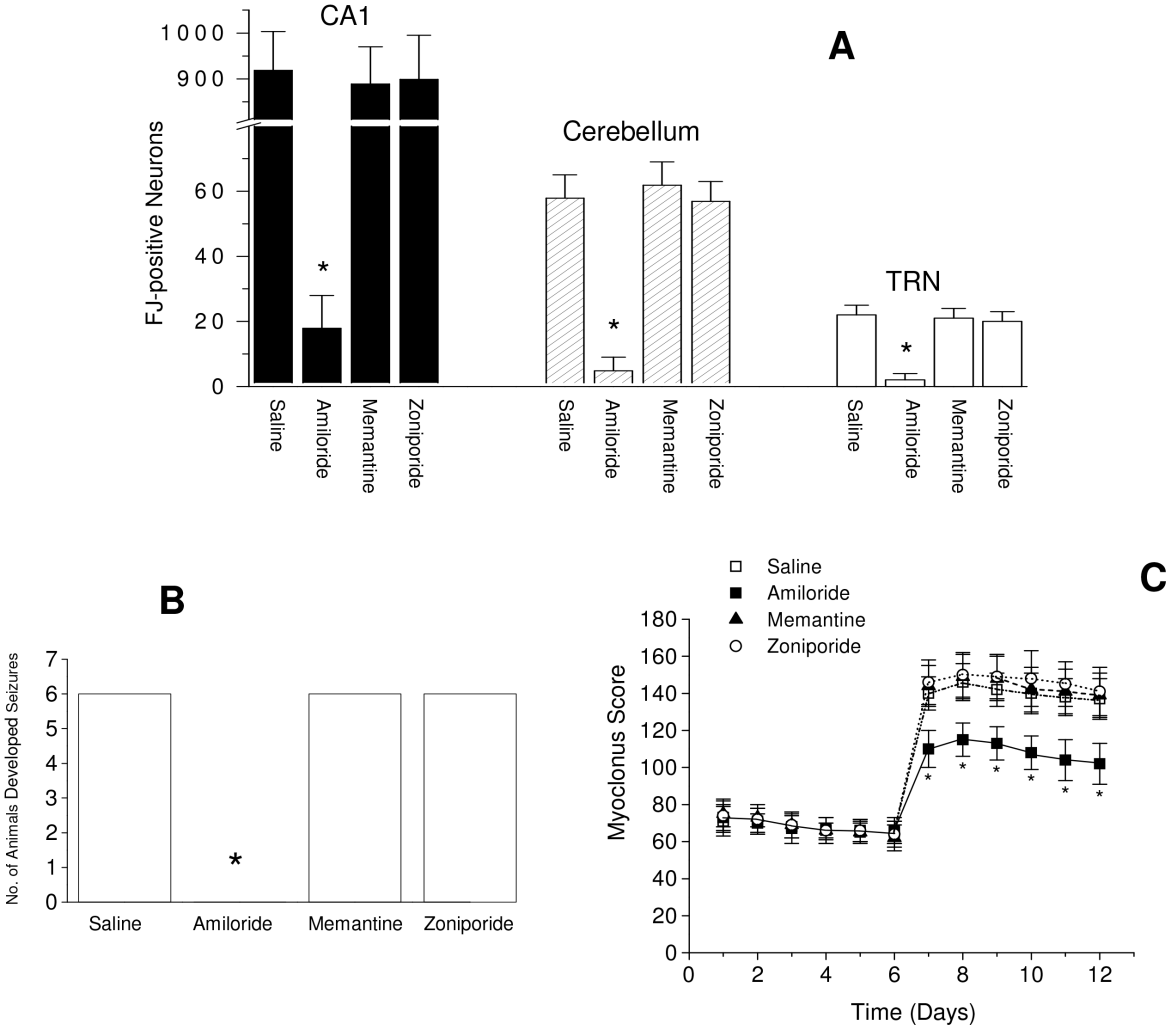
Memantine does not reduce cardiac arrest-induced cerebral hypoxic neurodegeneration, seizures and audiogenic myoclonic jerks when sodium pentobarbital was used as the anesthetic agent in this animal model. Rats were anesthetized with a single intraperitoneal injection of sodium pentobarbital (Nembutal) supplemented with a single subcutaneous injection of buprenorphine. (A) The number of FJ-positive degenerating neurons in the hippocampal CA1, the cerebellum and the TRN. (B) The number of posthypoxic rats that developed seizures. (C) The myoclonus scores recorded in the rats before and after cardiac arrest. The rats were received intracisternal injection of saline or 5 nmole of memantine, amiloride or zoniporide before cardiac arrest. * (*p*<0.05) indicates significant difference between the saline-injected group and the amiloride-pretreated group. Values are mean ± S.D., n = 6.

Because ketamine, the anesthetic agent used in this animal model, is itself a NMDA receptor antagonist, the neuroprotective effects of the drugs were also investigated with sodium pentobarbital (Nembutal) was used as the anesthetic agent. Intracisternal injection of memantine before cardiac arrest-induced cerebral hypoxia did not reduce cerebral hypoxia-induced neurodegeneration in the hippocampal CA1, the cerebellum and the TRN (Fig. 5A). Pretreatment with memantine also did not reduce the number of posthypoxic rats developed seizures (Fig. 5B). In addition, memantine did not reduce the audiogenic myoclonic jerks (Fig. 5C). These results confirm that NMDA receptors probably do not play a significant role in cerebral hypoxia-induced neurodegeneration, seizures and myoclonus.

## Discussion

Results from the present study showed that amiloride is very effective in protecting against cardiac arrest-induced cerebral hypoxic neurodegeneration in the hippocampal CA1; the cerebellum and the TRN. Amiloride is also effective in reducing seizures and audiogenic myoclonic jerks in the posthypoxic rats. In contrast, intracisternal injection of zoniporide in this animal model, did not alter cerebral hypoxia-induced neurodegeneration, seizures and audiogenic myoclonic jerks. These results suggest that blockade of ASICs rather than inhibition of the sodium-hydrogen exchanger is primarily responsible for the neuroprotective effect of amiloride against cerebral hypoxia-induced neurodegeneration and the neurological dysfunctions. A previous study has shown that inhibition of sodium-hydrogen exchanger may improve neurologic protection in a cerebral ischemia-reperfusion pig model of hypothermic circulatory arrest [24]. The deep hypothermic circulatory arrest model involves cooling the animal body and stopping blood circulation. In the cardiac arrest-induced cerebral hypoxia rat model used in the present study, the temperature of the animal body was maintained between 34–37°C by heat lamps and a circulating water warming pad. There are differences in the mechanism by which brain injury was introduced between these two animal models. In addition, in the deep hypothermic circulatory arrest animal model, injury is primarily caused by oxygen-derived free radicals, resulting in organ and endothelial dysfunctions [25]. The lack of neuroprotective effect by sodium-hydrogen exchanger inhibition in this cardiac arrest-induced cerebral hypoxia animal model may indicate that oxygen-derived free radicals may not play a crucial role in the cardiac arrest-induced hypoxic brain injury.

Amiloride has been reported to inhibit other exchangers including sodium-calcium exchanger [26]. Our results did not rule out the possibility that the observed neuroprotective effect of amiloride is due to inhibition of sodium-calcium exchanger. However, because efflux of cell calcium by sodium-calcium exchanger is considered a general mechanism for maintaining neuronal cell calcium concentration [27], inhibition of intracellular calcium extrusion through this exchanger by amiloride would lead to cell calcium overload and injury. Thus, inhibition of sodium-calcium exchanger by amiloride cannot explain the neuroprotective effect of amiloride observed in this study.

Pre-treatment with intracisternal injection of memantine under one dose, when sodium pentobarbital was used as the anesthetic agent, did not affect cerebral hypoxia-induced neurodgeneration, seizures and audiogenic myoclonic jerks. These results suggest that blockade of NMDA receptors do not protect cardiac arrest-induced hypoxic brain injury. The lack of neuroprotective effect from NMDA receptor antagonism in a pig model of cardiac arrest and resuscitation has been reported in the literature [28]. It has been known that lowering of the extracellular pH decreases the sensitivity of the glutamate receptors [29] which in turn decreases neuronal excitability and injury. It has also been shown that extracellular acidosis associated with global cerebral ischemia reduces the NMDA receptor activation and glutamate neurotoxicity in cortical cultures [30]. The absence of penumbra in global cerebral ischemia animal model in which lowering extracellular pH is probably one of the key conditions which does not favor triggering the NMDA receptor-mediated excitotoxic reaction cascade [31]. As a result, though glutamate is released during cerebral ischemia but NMDA receptors are blocked by extracellular acidosis, the NMDA receptor-mediated neuronal injury is thus prevented from happening during brain ischemia. Overall, our results provide new evidence to refute the notion that during cerebral ischemia, dysregulated glutamate release excessively activates NMDA receptors resulting in excitotoxic brain injury [4], [32], [33]. Our results implicate that NMDA receptors probably do not play a significant contributing role in global cerebral hypoxia-induced brain injury during ischemia.

Because amiloride is a potent blocker for the ASIC1a [34], the predominant ASIC member expressed in human brain [35], our results suggest that activation of this proton-gated cation channel by acidosis during cerebral hypoxia probably plays a pivotal role in cerebral ischemic brain injury. This could also be true in other global cerebral ischemia animal models as well. Indeed, emerging evidence from recent *in vitro* studies in human brain neurons [35] and in the rat glial cells [36] also showed that acidosis induced neuronal injury via the activation of ASICs. Results from the present study support this notion. Our results did not rule out the involvement of other natural ligands other than the proton ions in ischemic injury. A nonproton sensor for activating an ASIC family member has recently been identified [37], this finding implicates that ASICs might be activated by some natural nonproton activators during normal or pathophysiological conditions such as cerebral ischemia.

In this cardiac arrest-induced cerebral hypoxia and reperfusion animal model, cell injury can occur in two phases. During the hypoxic period, accumulation of lactic acid which lowers cellular pH which in turn activates ASICs allowing influx of extracellular sodium and calcium ions into the cells. High level of intracellular calcium activates phospholipase enzymes which damage membrane phospholipids, it also activates proteolytic enzymes which breakdown cytoskeleton of neurons. Restoring blood flow during reperfusion after eight minutes of hypoxia sets the stage for oxygen to generate free radicals including reactive nitrogen species such as nitric oxide and peroxynitrite which inflict tremendous membrane damage to neuronal, endothelial and blood cells. This disrupts blood brain barrier and exacerbates brain damage. Nitric oxide is also known to be capable of potentiating ASICs activity and thus induce further cell injury.

In addition to its neuroprotective effects, amiloride significantly reduced the number of posthypoxic rats to develop seizures and reduced the severity of audiogenic myoclonic jerks. In contrast, memantine did not alter the number of the posthypoxic animals to develop seizures. Instead, memantine might slightly elevated audiogenic myoclonus severity in the posthypoxic rats, this result is consistent our recent finding that memantine exacerbated myoclonic jerks of the posthypoxic animals when it was administered intraperitoneally [21]. Results from previous studies by others have shown that systemically administration of amiloride has anticonvulsant activity [38], [39] in *in vivo* models of seizure and that amiloride can also enhance the anticonvulsant activity of a number of antiepileptic drugs [40]. Because amiloride cannot get pass the blood brain barrier due to its hydrophilic nature, whether these observed anticonvulsant effect of amiloride was related to its action in the central nervous system is not clear. But the antiseizure effects and the potent neuroprotective effects of central administration of amiloride as shown in the present study suggest that ASICs could be a molecular target for the development of antiepileptic agents. Because amiloride is far more effective in reducing global cerebral hypoxia-induced neurodegeneration than reducing seizures and posthypoxic myoclonus, this may implicate that the neuronal injuries in the hippocampal CA1, the cerebellum, and the TRN may play a limited contributing role in the genesis of seizures and myoclonus, injury in other areas of the brain may be involved in the genesis of seizures and myoclonus.

In conclusion, our results suggest that ASICs may play a more pivotal role than NMDA receptors in mediating cardiac arrest-induced global cerebral hypoxic neurodegeneration. ASICs rather than NMDA receptors is a potential molecular target for the development of treatments and therapeutic interventions for ischemic brain injury.
